# Rendering algorithms for aberrated human vision simulation

**DOI:** 10.1186/s42492-023-00132-9

**Published:** 2023-03-17

**Authors:** István Csoba, Roland Kunkli

**Affiliations:** 1 Faculty of Informatics, University of Debrecen, Debrecen 4028, Hungary; 2 Doctoral School of Informatics, University of Debrecen, Debrecen 4028, Hungary

**Keywords:** Human vision, Human visual system, Vision simulation, Wavefront aberrations, Visual aberrations, Vision-realistic rendering

## Abstract

Vision-simulated imagery―the process of generating images that mimic the human visual system―is a valuable tool with a wide spectrum of possible applications, including visual acuity measurements, personalized planning of corrective lenses and surgeries, vision-correcting displays, vision-related hardware development, and extended reality discomfort reduction. A critical property of human vision is that it is imperfect because of the highly influential wavefront aberrations that vary from person to person. This study provides an overview of the existing computational image generation techniques that properly simulate human vision in the presence of wavefront aberrations. These algorithms typically apply ray tracing with a detailed description of the simulated eye or utilize the point-spread function of the eye to perform convolution on the input image. Based on the description of the vision simulation techniques, several of their characteristic features have been evaluated and some potential application areas and research directions have been outlined.

## Introduction

Vision is one of the main mechanisms used to perceive the world. By capturing the light reflected from the surrounding objects, the brain can gather a wide range of information and support a variety of tasks in everyday lives.

The organs that enable vision are the brain and human eye. The human eye acts as an optical system and is responsible for collecting incoming light rays. It is a slightly ovoid organ comprising the cornea and crystalline lens as its main refractive components. The incoming light rays pass through these elements and focus on the back of the eye (the retina), where millions of light-sensitive photoreceptors (the rods and cones) are activated to sense this light. The information thus collected is then forwarded to the brain, which performs various postprocessing tasks to correct the optical limitations of the eye, generating a final image as output, referred to as vision.

The performance of the human eye is heavily affected by a variety of factors despite its robust optical design [1]. Optical limitations of the eye are a typical source of visual imperfection that can be characterized by wavefront or visual aberrations. Wavefront aberrations describe the deviations that the optical system causes in light wave paths as light passes through the refractive elements of the system. Measuring these aberrations using clinical devices (wavefront aberrometers) is a common ophthalmic process in the study of visual aberrations for the characterization of the visual acuity of the eye.

**Fig. 1:**
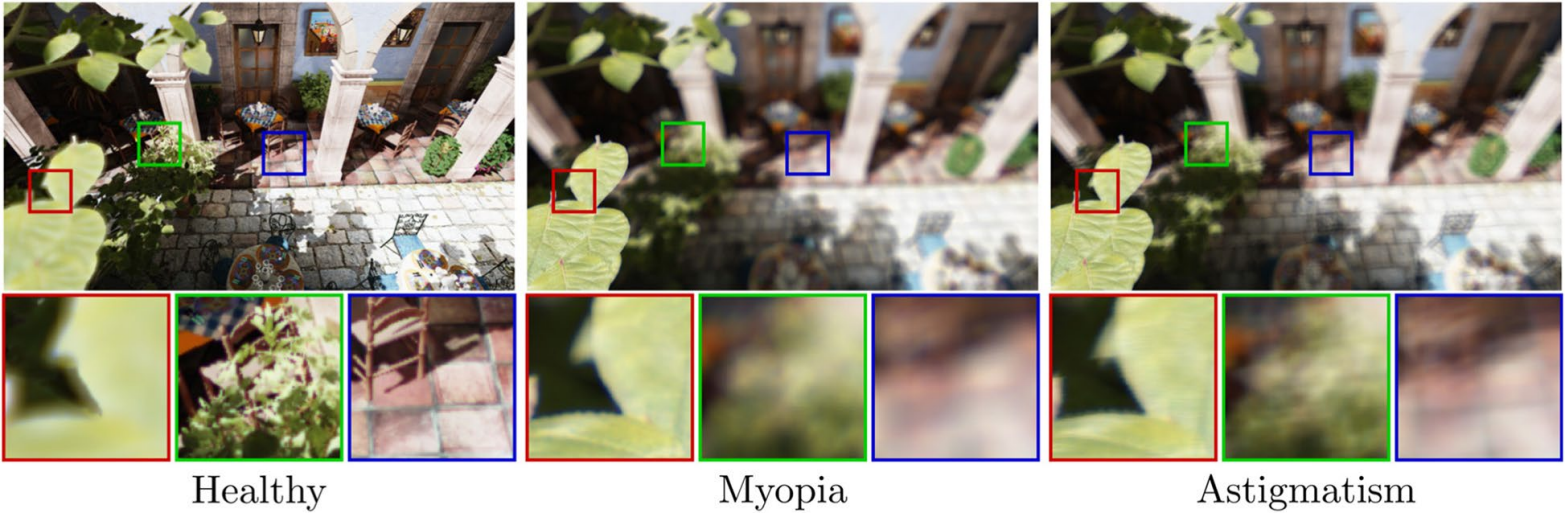
Comparison of three common eye conditions. The visual acuity and the overall characteristics of these eyes differ significantly, which is the main reason why vision-simulated imagery is an important tool of ophthalmology

An important and highly unfortunate aspect of visual aberrations is that they affect every living person; this is because the eye’s inherent complexity causes some aberrations even in otherwise healthy vision [2]. In addition, wavefront aberrations induced by external factors have considerable impact on a large portion of the population. Such effects are a natural result of aging and exposure to the outside world, with common causes that include deformation of the cornea, stiffening of the crystalline lens, and changes in the axial length of the eye. Consequently, aberrations are unique to each person and eye condition, as shown in Fig. 1. Thus, vision simulation is invaluable and has many potential applications in ophthalmology [3–6].

The problem of simulating the performance of an optical system is not new in the field of computer graphics. A prominent component of plausible visual simulations is the proper use of the depth-of-field (DOF), for which researchers have developed a large set of different rendering algorithms [7]. Most existing vision simulation methods draw inspiration from these techniques and extend them with interesting new ideas to address the complexity and intricacies of the human visual system. This study provides an overview of these techniques, focusing on the optical simulation of human vision in the presence of wavefront aberrations.

## Vision simulation using ray tracing

Ray tracing for image synthesis has a long history in computer graphics. It is a technique that utilizes the rays recursively bouncing around in the scene to collect color information through the intersection of these rays with objects and light sources. A large number of ray-tracing approaches have been proposed to address the computational complexity of the problem in an efficient manner. These methods are mainly categorized as forward ray tracing (the rays are traced from the light source to the objects in the scene through the optical system and then captured on the image sensor) and backward ray tracing (the rays are traced outwards from the image plane through the optical system to the objects and light sources in the scene). However, there also exist hybrid algorithms that combine the advantages of these two approaches. Covering the details of these algorithms is beyond the scope of this survey. The reader is referred to ref. [8] for a more elaborate introduction into the inner workings of a ray tracer.

Several studies have utilized ray tracing for human vision simulations. Table 1 provides an overview of the studies discussed in this section. In the following section, the main aspects of vision simulation using ray tracing is outlined.

**Table 1:** Summary of the methods discussed in this study that utilize ray tracing for vision simulation

Reference	Method used and main innovation	Main limitation
Mostafawy et al. [9]	The authors used distributed ray tracing with 3D scenes. The eye was modeled using spherical refractive elements and a flat retina, and optionally extended by corneal refractive zones. Focusing was achieved by modifying the crystalline lens parameters	No aspherical elements were used, the flat retina caused incorrect peripheral blurring, and no spectral tracing was performed
Fink and Micol [10]	This work used Zernike polynomials to represent the refractive eye elements, distributed ray tracing with 2D images as input, and an inverse ray-tracing procedure with an emmetropic eye model to unwarp the retinal images	The intersection tests were very costly, the input was limited to 2D images, and chromatic aberration was ignored
Wu et al. [11]	The authors used aspherical refractive eye elements to improve the peripheral simulation accuracy and bidirectional path tracing with 3D scenes to guarantee that no rays are blocked by the pupil	Chromatic aberration was ignored and the flat retina shape caused incorrect peripheral blurring
Wang and Xiao [12]	This algorithm utilized spectacle lens prescriptions for a fast simulation of myopia, hyperopia, and astigmatism, and a novel simplified geometrical formulation for a stripped-down eye model to efficiently calculate the refracted ray directions	The supported aberration types were very limited and chromatic aberration was ignored
Wei et al. [13]	This method used distributed ray tracing with 3D scenes and a triangle-based human eye model to significantly improve the computational performance of ray-intersection tests	The intersection tests were overall still costly compared to the closed-form analytical approaches
Dias et al. [14]	The authors examined the effects of multiple retina shapes using distributed ray tracing with 3D scenes and the Navarro schematic eye model	Chromatic effects were ignored and the resulting retinal images were not unwarped
Cholewiak et al. [15]	The authors simulated chromatic aberration by modeling the human eye using a lens with a wavelength-dependent focal length	The minimalistic eye model could not simulate other types of aberrations
Lian et al. [16]	The authors described an open-source software package that used distributed ray tracing with 3D scenes and aspherical eye models and simulated diffraction, spectral effects, and cone excitations	The resulting retinal images were not unwarped
Vu et al. [17]	This work used forward ray tracing with 2D images and aspherical eye elements, and a novel GPU-based rasterization approach that substantially reduced the noise levels resulting from ray tracing	Chromatic aberration was ignored and the simulation was limited to 2D images

### Distributed ray tracing

**Fig. 2:**
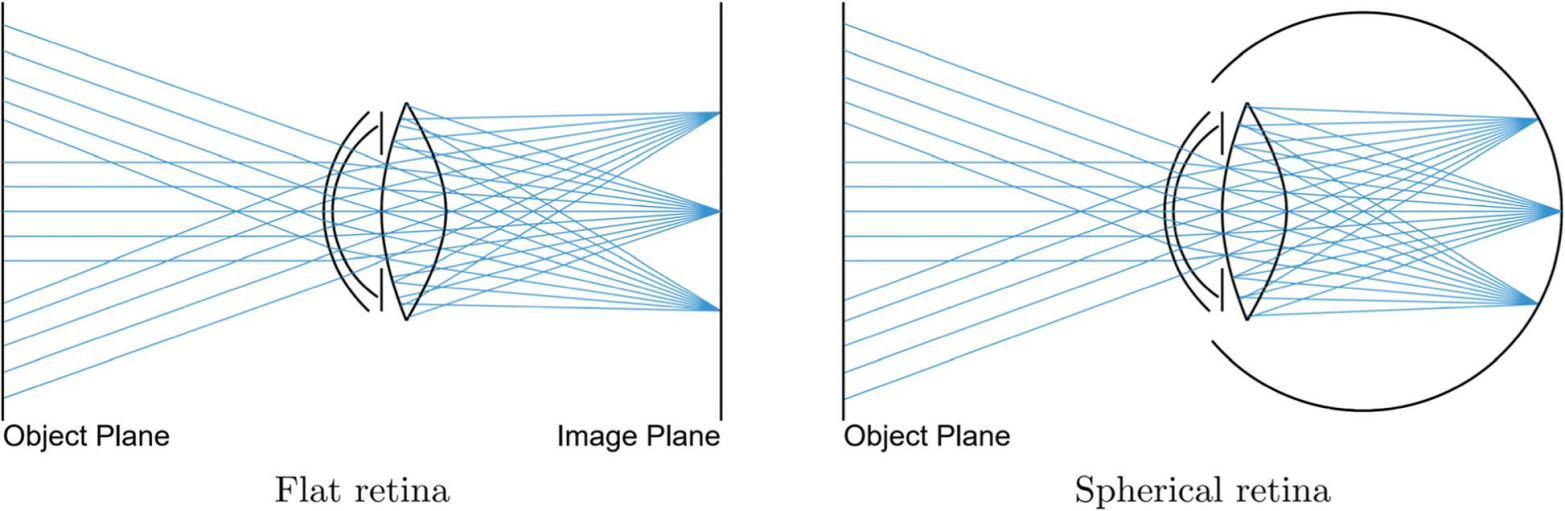
Schematic of distributed ray tracing. Rays starting from the retina are traced through the eye, sampling the back surface of the crystalline lens to determine their outgoing directions. Mostafawy et al. modeled the retina as a flat surface (left), whereas more recent approaches often use a spherical representation (right)

The first method for simulating human vision via ray tracing was introduced by Mostafawy et al. [9]. Their image formation approach utilizes distributed ray tracing [18] combined with analytical surface descriptions to model the various elements of the optical system [19]. The simulated image is constructed by averaging the contributions of a bundle of rays traced through the optical system, which is performed for every output pixel. Each ray is launched toward a different point on the anterior lens surface (the back of the crystalline lens), and thus samples a different point from the physical pupil disk. This process is generally referred to as backward ray tracing and is illustrated in Fig. 2. The quality of the simulation is determined mainly by the number of per-pixel rays traced.

Regarding the description of the eye, the approach of Mostafawy et al. [9] is built on the Gullstrand eye model that uses spherical surfaces to model the refractive elements of the eye. To simulate focusing and low-order aberrations, the model was extended with scaling terms and modifiable curvatures and corneal refractive zones were employed to simulate the impact of photorefractive keratectomy on the subject’s vision.

### Spherical retinas

In the approach proposed by Mostafawy et al. [9], the image plane is assumed to be flat, which fails to properly account for curved retinas. Consequently, the peripheral areas of the resulting simulations are significantly over blurred.

Dias et al. [14] circumvented this limitation by employing a spherical retina shape and replacing the Gullstrand model with the wide-angle aspherical Navarro eye model. Figure 2 displays a comparison of the ray-tracing process on two retina shapes.

The final simulation is then obtained by projecting the resulting spherical image onto the image plane using an orthographic projection matrix. Alternatively, Fink and Micol [10] demonstrated that an idealized eye model (i.e., one with minimal aberrations) can be used to unwarp the resulting images.

### Efficient sampling

**Fig. 3:**
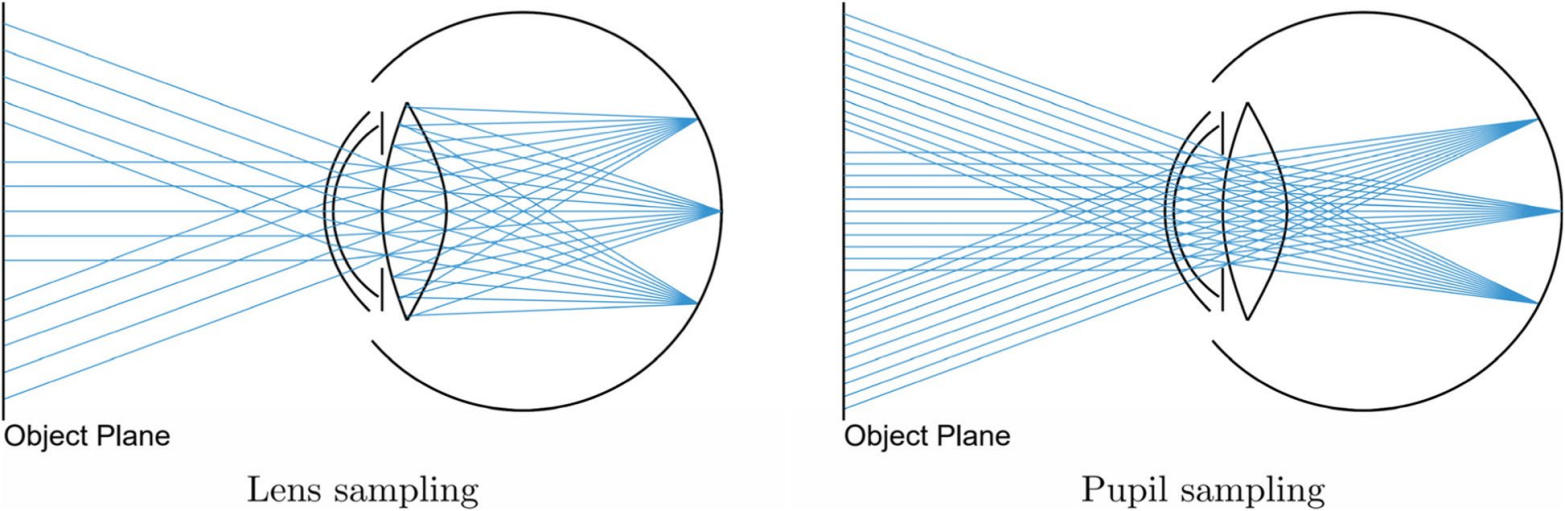
Comparison of strategies for determining the outgoing ray directions. Sampling the back of the lens leads to many rays being blocked by the pupil (left), whereas every ray can trace through the whole eye unblocked by utilizing pupil sampling instead (right)

The distributed ray-tracing algorithm described above randomly samples the anterior lens during the construction of ray bundles. Such an approach can potentially lead to a large number of rays being blocked by the pupil, as shown in Fig. 3. Consequently, the resulting image quality can be severely degraded unless a large number of rays are cast for each output pixel.

To overcome this issue, Wu et al. [20] proposed the use of a ray-tracing method, which is an extension of the bidirectional path-tracing algorithm [21]. They demonstrated the applicability of their approach using multiple schematic eye models, with each comprising spherical and aspherical elements [11].

The gist of their solution is to sample the entrance pupil directly during the ray-generation step, which guarantees that no sample is blocked by the physical pupil. This is called a camera subpath. Rays are also initiated from the light sources of the scene in the light subpath. The algorithm then attempts to connect the various hits along these two subpaths. If an unblocked connection can be made, the starting location of the ray on the entrance pupil is traced through the eye model to find the corresponding image-space projection. Such a sampling strategy can also be combined with distributed ray tracing, as shown in Fig. 3.

### Chromatic aberration

Chromatic aberration results from the wavelength-dependent refraction index of the optical elements of the eye, leading to a different focus distance per wavelength of light. Cholewiak et al. [15] studied the impact of chromatic aberration on accommodation and depth perception and concluded that wavelength-related cues are important elements of both processes. They modeled the refractive elements of the eye with a simple finite-aperture lens and simulated the spectral effects using a wavelength-dependent focal length.

**Fig. 4:**
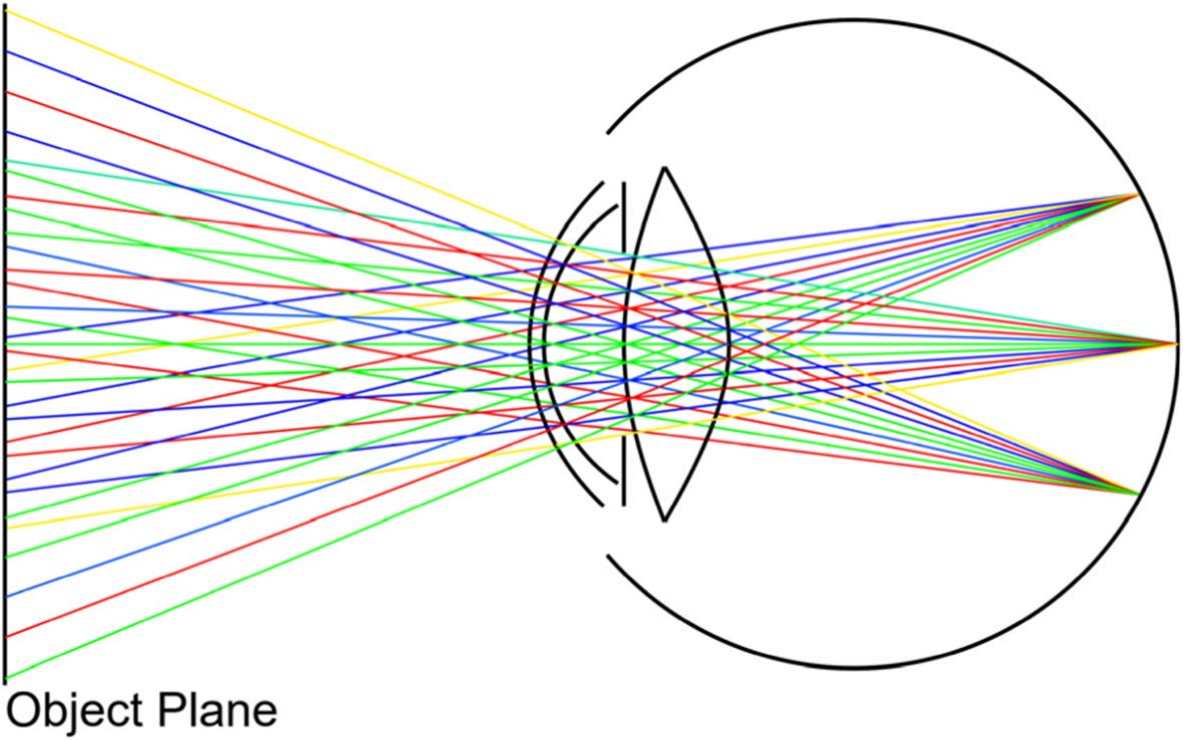
Overview of spectral ray tracing. Each outgoing ray is assigned a different wavelength, which is used to calculate the wavelength-dependent refraction indices during ray tracing

A different yet more physically correct approach of simulating chromatic aberration is to assign a different wavelength to each ray and compute the wavelength-dependent refraction index of the simulated optical elements during ray tracing. This approach was adopted in the algorithm of Steinert et al. [22], which was also utilized by Lian et al. [16] in their vision simulation framework called ISET3d. A simplified visualization of the algorithm is shown in Fig. 4. In this approach, spectrum sampling is typically performed uniformly for the three RGB channels or randomly, however, both approaches lead to a significant increase in the number of rays necessary to keep noise levels low.

### Diffraction effects

The distributed ray-tracing approach described thus far ignores the wave-related effects. Although the effect of diffraction is generally small with large pupil diameters, the severity of its impact increases as the pupil size decreases. Therefore, not considering these effects can significantly reduce the quality of the simulation.

**Fig. 5:**
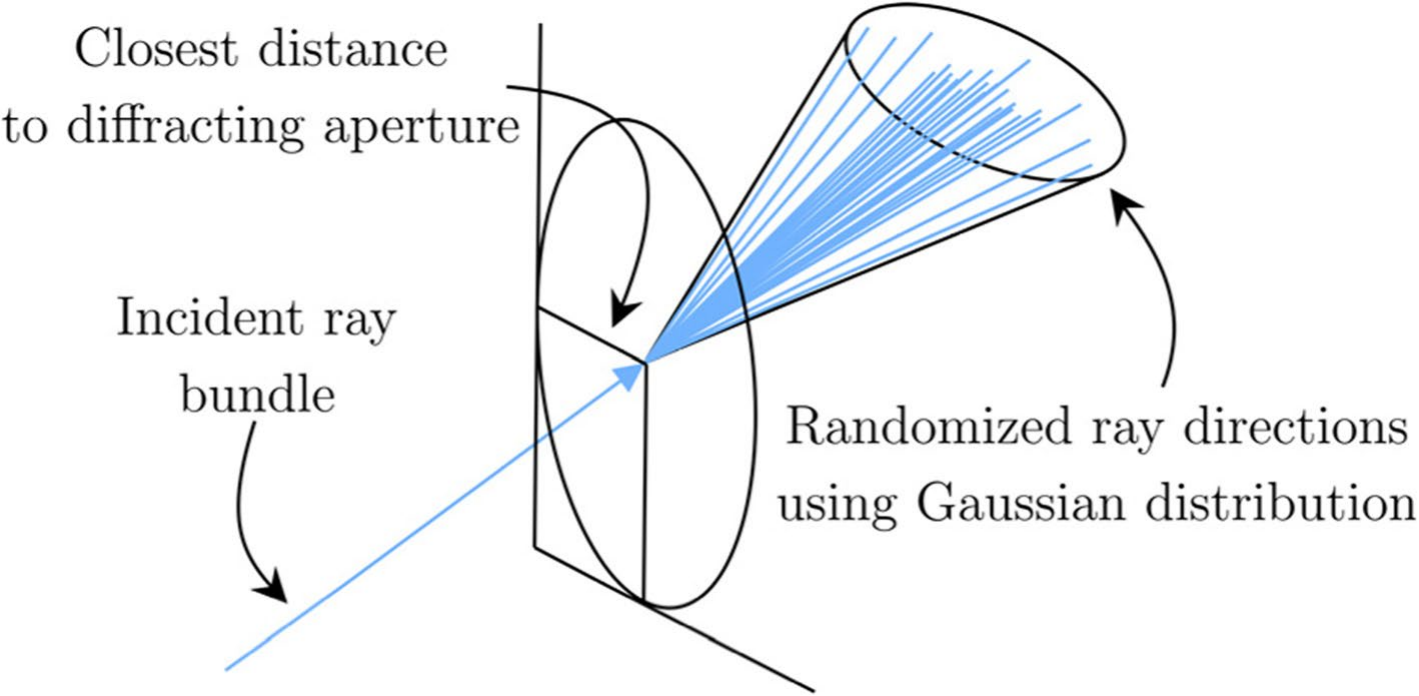
Diffraction simulation using the HURB method. The outgoing ray directions are perturbed using a Gaussian distribution when they pass through the pupil. The amount of scattering depends on the distance from the edge of the pupil

To simulate diffraction, the Heisenberg uncertainty ray bending (HURB) method [23] was employed by Lian et al. [16] in their ISET3d framework. The HURB method randomizes the direction of the rays as they pass through the pupil. A schematic of this approach is shown in Fig. 5. The probability distribution function is a bivariate Gaussian function, where the variance (angle of scattering) increases toward the edge of the pupil. Such a formulation leads to more blurring when the pupil size decreases because an increasing number of rays originate close to the edge of the pupil. The tradeoff of this approach is an increase in the number of rays and per-ray computation time.

### Reducing trace complexity

All the ray-tracing algorithms described above model the eye using a complex system of spherical or aspherical elements. Although such a description has several benefits (such as the ability to support a wide range of eye conditions and chromatic aberration), it also contributes to lengthy computation times; this makes the process of simulating simple eye aberrations unnecessarily complex.

To overcome these issues, Wang and Xiao [12] used a simple eye model comprising a convex lens, pupil, and flat retina. This stripped-down model allows for the derivation of simple geometrical formulas for eyes with myopia, hyperopia, and astigmatism. These formulas are much more efficient than full-blown ray tracing with intersection tests and refractions. The main limitation of this approach is that the supported types of aberrations are very limited and the algorithm ignores all the aspects of vision simulation (chromatic aberration, non-flat retinas, etc.) described above.

### Complex surface descriptions

In contrast to the previous case, there are situations where the main concern is quality and not the cost of ray tracing. While the aspheric surfaces and multiple refractive zones in the previous techniques can be used to simulate a wide range of eye conditions, the model is not detailed enough to accurately capture every nuance of a real human eye.

To construct a model that can simulate arbitrary conditions, the use of the circle polynomials of Zernike [24] was proposed by Fink and Micol to describe the various optical elements of the human eye [10]. The Zernike polynomials are a set of orthogonal polynomials over the unit circle and are commonly used in the ophthalmic literature. A least-squares fitting procedure [25] was utilized by the authors to obtain the coefficients necessary for modeling the refractive surfaces and employed backward ray tracing to obtain the final image. To circumvent the complexity of the surfaces thus constructed, the necessary intersection points were calculated using the iterative Newton–Raphson method [26].

**Fig. 6:**
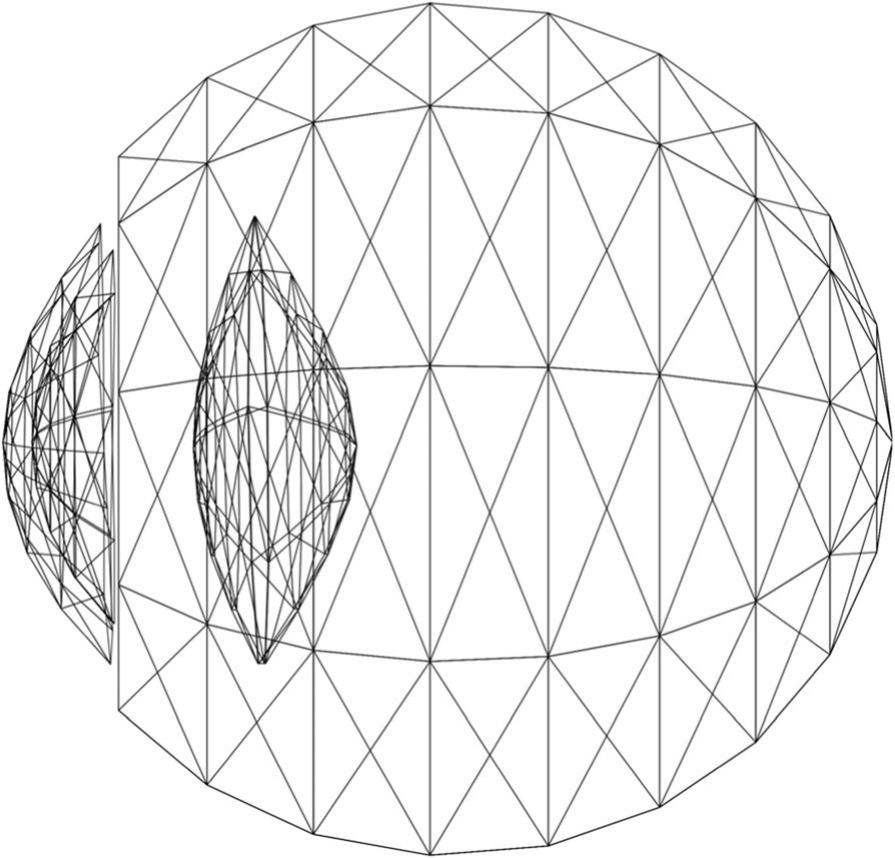
An example eye model suitable for the algorithm of Wei et al., where each diffracting surface is modeled using triangles. Such a representation facilitates the calculation of intersection points using triangle-based tools instead of the more expensive analytical formulas

The main disadvantage of this approach is the cost of calculating intersection points. The iterative nature of the Newton–Raphson method and the complexity of evaluating surface points, both play an important role in this problem. The use of polygon meshes was proposed by Wei et al. [13] to increase ray-tracing speed. An example of a polygon mesh-based eye model is shown in Fig. 6. Such an approach retains the ability to model arbitrary eye conditions and facilitates the application of modern tools dedicated to ray tracing with polygon meshes. Combined with backward ray tracing, their approach yielded substantial improvements in computational performance.

### Reducing noise

The stochastic nature of distributed ray tracing introduces noise into the resulting simulations. The impact of noise can be reduced by increasing the number of rays traced per pixel; however, this approach causes an inordinate increase in computation times. Denoising [27] is another common form used to drastically improve the quality of renderings with low per-pixel sample counts but requires an extra nontrivial step on top of the existing rendering pipeline.

**Fig. 7:**
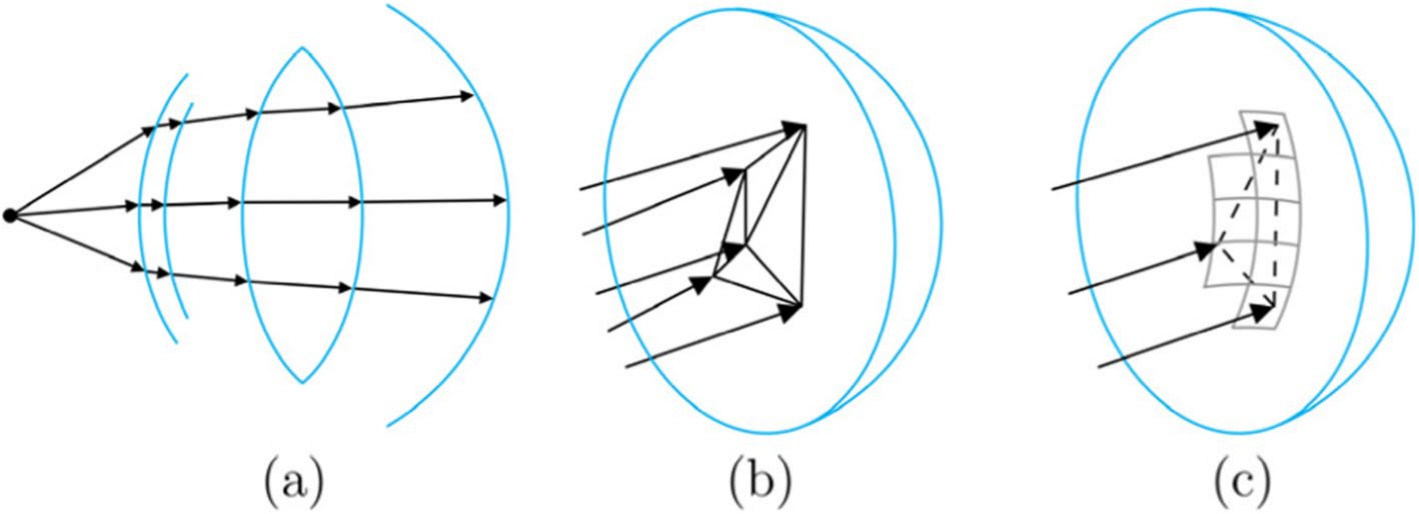
Schematic of the forward ray-tracing approach of Vu et al. **a** An incoming ray bundle is traced through the optical system; **b** The resulting retinal points are used to form a triangular grid; **c** The triangles are rasterized onto the output image

To overcome this issue, Vu et al. [17] proposed a different method that combines forward ray tracing with triangulation, similar to the lens flare-rendering algorithm of Hullin et al. [28]. An overview of this method is presented in Fig. 7. The algorithm of Vu et al. uses a uniform grid of rays originating from each light source, which was traced toward the entrance pupil of the eye. After the rays successfully trace through the eye and hit the retina, the neighboring rays are used to construct a set of triangles. These triangles are then manually rasterized onto the resulting image to generate vision-simulated imagery. This approach guarantees that the resulting images have no gaps between the ray-traced samples, leading to smooth and noise-free outputs.

## Image-space approaches

In addition to ray tracing, another main method for visual aberration simulations is via postprocessing filters that operate on prerendered images. Performance often being a key factor, these approaches are usually employed in rasterization-based systems. Rasterization works by taking a set of input primitives (typically in the form of triangles in 3D space) and determining the list of image pixels that each primitive covers. Per-object transformations and camera projections are handled by transforming the vertices of the input primitives using 4 × 4 transformation matrices. A pinhole camera model is often assumed, with physical camera-based effects simulated via postprocessing. Correct depth ordering is usually resolved using a per-pixel depth buffer, which outputs a depth map, and is often utilized by image-space filters. For a more elaborate explanation of rasterization-based image synthesis, the reader is referred to ref. [29].

Many algorithms operate in the image space to produce vision-simulated images. Table 2 provides an overview of the studies discussed in this section. The following section covers several important aspects of these methods.

**Table 2:** Summary of the image-space human vision simulation methods discussed in this study

Reference	Method used and main innovation	Main limitation
Camp et al. [30]	The authors utilized paraxial ray tracing with corneal topography measurements to compute the on-axis point-spread function (PSF) of the human eye and simulated vision by convolving 2D images with the resulting PSFs	The input ignored the internal aberrations of the eye and the paraxial approach suffered from accuracy issues
Greivenkamp et al. [31]	This work used exact ray tracing with a schematic eye model to calculate the on-axis PSF of the eye and modeled the Stiles-Crawford effect using an apodizing filter	The simulation was limited to 2D images and peripheral vision was ignored
Rokita [32]	The author used repeated filtering with a simple 3 × 3 kernel to approximate the depth-dependent blur of the human eye and utilized the focus distance and input focal length to determine the per-pixel amount of blurring	The lack of real eye information limited the supported types of eye conditions
Barsky [33]	This work used wavefront aberrations to calculate the depth-dependent, on-axis PSFs of the eye and split the input images into depth-dependent slices for convolution with the PSFs	The depth slices had banding artifacts and chromatic aberration and peripheral vision were ignored
Rodríguez Celaya et al. [34]	The authors simulated progressive lenses using sparse, 3D PSF grids (with different axes corresponding to the horizontal angle, vertical angle, and depth), which were interpolated on a per-pixel basis during convolution	The PSF grid was too sparse, the range of incidence angles was limited, and chromatic aberration was ignored
Kakimoto et al. [35]	This algorithm simulated vision through progressive lenses by rendering the scene from multiple views using a precomputed 3D map to compute the per-vertex displacement of each view	The simulation was limited to low-order aberrations and performance scaled poorly with scene complexity
Kakimoto et al. [36]	This work extended the previous multiview method [35] by using conoid tracing to reduce the length of precomputation	This method exhibited the same main limitations as the previous approach [35]
Barsky [37]	The author solved the artifacts of their previous slice-based approach [33] using edge detection to ensure that objects spanning multiple slices are fully included in all slices	Peripheral vision and chromatic aberration were not simulated
Watson [38]	The author used Zernike aberration coefficients and the Fourier transformation to efficiently compute the PSFs of the human eye for varying pupil sizes and object distances	Vision simulation was limited to a single object plane
Tang and Xiao [39]	This work simulated low-order eye aberrations in real-time using an elliptical Gaussian kernel and per-pixel blur field to support peripheral vision and variable eye parameters	Higher-order aberrations (HOA) and chromatic effects were not supported
Barbero and Portilla [40]	The authors used local dioptric matrices to simulate vision through progressive lenses at different gaze directions and approximated the PSFs using samples placed on an ellipse	The inherent eye aberrations and per-pixel depth information were ignored
Cholewiak et al. [41]	This work computed human PSFs by properly simulating longitudinal chromatic aberration	Per-pixel depth information and peripheral vision were ignored
Gonzalez Utrera [42]	The author presented an improved PSF interpolation method for off-axis PSFs and utilized depth-dependent slices to convolve 3D scenes	The PSF grid was too coarse to properly simulate off-axis vision and the slicing caused banding
Csoba and Kunkli [43]	This work used spectacle lens prescriptions to simulate low-order aberrations in real-time environments by utilizing separable complex kernels to approximate the PSFs	HOA were not supported and chromatic aberration and peripheral vision were ignored
Csoba and Kunkli [44]	The authors estimated the physical eye structure from aberrations to compute a coarse PSF grid for varying parameters and simulated vision with an approximately real-time performance profile using tiled convolution and a novel GPU-based per-pixel PSF interpolation approach	The precomputation step was long, peripheral vision was ignored, and partial occlusion was not handled
Lima et al. [45]	This work simulated low-order aberrations using light-gathering trees to efficiently compute refracted light directions for samples on the pupil disk and handled partial occlusion using layered inputs	The simulation was limited to low-order aberrations and peripheral vision was not considered

### PSF-based convolution

**Fig. 8:**
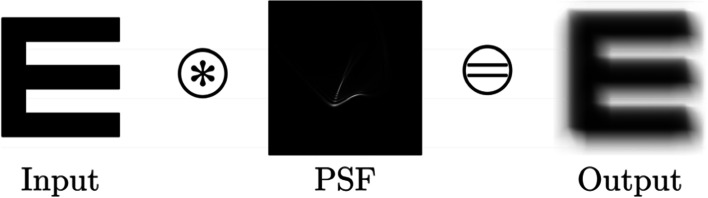
Vision simulation using PSF-based convolution

One of the earliest image-space approaches simulating visual aberrations was based on convolution with the PSF of the eye, which is the diffraction pattern of an ideal point source. Given the PSF, the eye can be treated as a black box, and vision can be simulated as the superposition of every object point modulated by the PSF of the optical system. This process is illustrated in Fig. 8.

Therefore, devising a method for the computation of the PSF is necessary. Even with the goal of simulating vision using convolution, ray tracing can still be used to obtain the PSF. Paraxial ray tracing with a measured corneal topography map was used by Camp et al. [30] to obtain the PSF. In this approach, a single ray originating from a fixed distance along the optical axis is traced against each measured point on the corneal surface. The rays are then refracted according to the local optical power and extended to the image plane. The resulting PSF was then used to perform convolution with a simplified Snellen eye chart.

Although the approach by Camp et al. was an important milestone for convolution-based vision simulation, it has several limitations. Specifically, the simplified eye model removes the inherent aberrations of the eye, which is further exacerbated by the inability of the paraxial approach to correctly simulate wavefront aberrations and diffractions. Greivenkamp et al. [31] demonstrated that the aforementioned problems can be solved using exact ray tracing and proper eye models that comprise aspherical elements. Moreover, the Stiles-Crawford effect [46] was considered in this model by utilizing a Gaussian falloff function centered on the entrance pupil.

### Multiple object distances

The two convolution-based approaches described above focus on 2D images. Although the ability to simulate a single object plane holds tremendous value, extending the approach to cover the entire focal region could provide a more holistic image of the individual’s vision. Furthermore, such an extension could also facilitate the simulation of 3D scenes and open the door for creating fully immersive simulations.

**Fig. 9:**
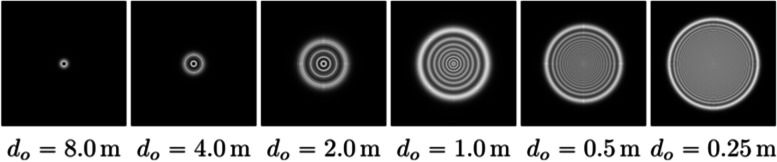
Through-focus PSFs for six different object distances

Motivated by these reasons, Barsky [33] introduced the concept of object-space point-spread function (OSPSF), which is a depth-dependent version of the traditional PSF. An example of a set of depth-dependent PSFs is shown in Fig. 9.

Similar to previous works, Barsky used ray tracing to compute the OSPSFs. However, instead of utilizing the eye model directly, Barsky proposed using wavefront aberrations of the eye to calculate perturbed ray directions. In their work, aberration functions are obtained using a Shack-Hartmann wavefront sensor, allowing vision to be simulated without measuring the physical properties of the eye.

Given a set of OSPSFs, vision is simulated by partitioning the input rendering into distinct depth regions and then convolving each slice with the OSPSF corresponding to its central depth value. This approach has a disadvantage—if an object spans several different depth regions, the convolution process may include discarded pixel values along the borders of the depth regions. Barsky referred to this phenomenon as a discretization artifact. To eliminate these artifacts, edge detection can be employed for object identification [37]. By including every object in the scene in all relevant depth layers, it is ensured that no incorrect values are used during convolution.

### Faster PSF computation

If the wavefront aberrations of an optical system are known, the PSF can also be computed using diffraction theory. To this end, Watson [38] used the Fraunhofer diffraction formula [47] as a more computationally efficient approach than ray tracing because it facilitates the computation of the PSF via the fast Fourier transform of the complex-valued generalized pupil function.

More recently, Csoba and Kunkli [44] proposed the use of the extended Nijboer-Zernike (ENZ) diffraction theory [48] as an alternative means of computing human PSFs for vision simulation purposes. The ENZ approach is based on the more accurate Debye-Wolf diffraction integral [49] and defines the PSF as a linear combination of a set of independent functions. Such a representation of the PSF not only enables computation with arbitrary precision but also facilitates the reuse of the linearized terms, yielding substantial performance benefits. They also proposed an efficient graphical processing unit (GPU)-based PSF computation method using the ENZ approach [50], which further improves the performance of the PSF computation step and facilitates the interactive computation of the necessary PSF kernels.

### Efficient convolution of 3D scenes

All the image-space methods described above used the convolution theorem (convolution in the frequency domain) to produce the final images. Although such an approach works well with 2D inputs or depth-based partitioning, it fails to accurately represent the spatially varying nature of the PSF. Furthermore, processing multiple slices is prohibitively expensive in interactive environments.

**Fig. 10:**
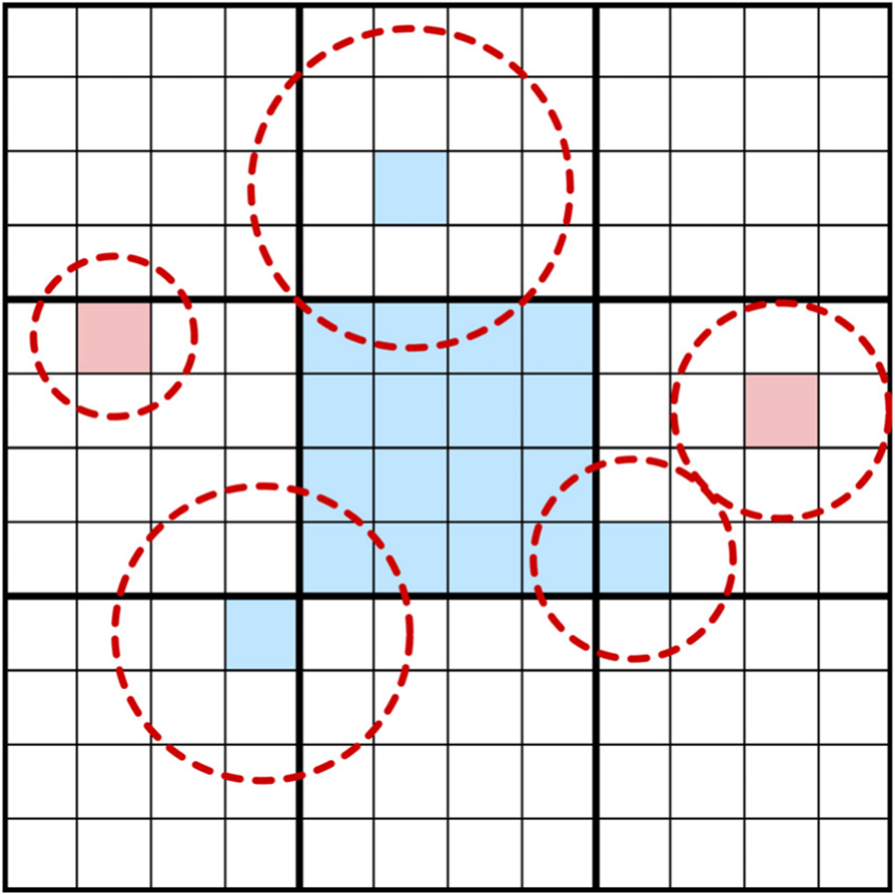
Collecting overlapping pixels for the middle tile by the tiled convolution algorithm and a set of example samples. The dashed circles represent the PSFs of the pixels and the pixel colors indicate the inclusion of the pixel in the tile buffer

To overcome these limitations, Csoba and Kunkli [44] utilized tiled splatting [51] with a GPU-based PSF interpolation technique to simulate visual aberrations at approximately real-time frame rates. Instead of relying on the convolution theorem, their approach performs a convolution directly in the spatial domain. The input image is split into independent tiles, and a set of per-tile buffers is constructed out of all pixels with overlapping PSFs, as shown in Fig. 10. The tile buffers are then sorted by depth for proper occlusion handling. The final image is produced by traversing the sorted per-tile buffers for each output pixel and accumulating the pixel contributions that are weighted by approximations of the dense per-pixel PSF.

To produce a dense PSF, their approach builds on Barsky’s concept [37]. The PSFs are computed for a small set of distinct object distances and stored in a GPU-accelerated texture. This texture is then used to approximate the PSF at any object distance via linear interpolation of the neighboring precomputed PSFs. This approach was validated by comparing their results to those obtained using convolution with the true dense PSF, and it was demonstrated that the outputs of their interpolation-based solutions are nearly identical to the ground-truth images.

### Chromatic aberration

**Fig. 11:**
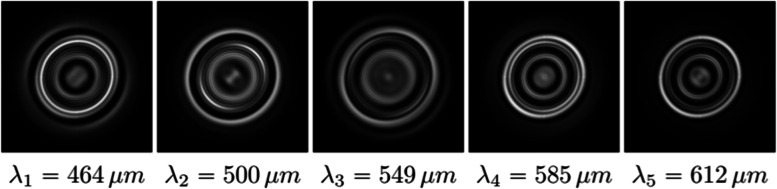
Chromatic PSFs for five different wavelengths

As described in the subsection on chromatic aberration (Vision simulation using ray tracing), chromatic aberration stems from the wavelength-dependent refractive index of the eye. As a result, the PSF of the eye also depends on the wavelength of light. An example of a set of chromatic PSFs is shown in Fig. 11. Consequently, chromatic aberration can be modeled by employing a wavelength-dependent PSF, which is usually performed in practice using one kernel for each channel of the input RGB images.

The chromatic PSF can be computed in multiple ways. In the case of ray-traced PSFs, the same strategies described in the Vision simulation using ray tracing section, can be employed. The situation is somewhat more complicated with diffraction theory, in which the wavelength-dependent aberration coefficients need to be obtained to compute the corresponding PSFs. Watson [38] and Cholewiak et al. [15, 41] demonstrated that this problem can be solved using empirical formulas to derive the amount of defocus introduced by chromatic aberration.

Alternatively, if the eye structure is available, aberration coefficients can also be obtained using ray tracing and least-squares coefficient fitting, the applicability of which was demonstrated by Csoba and Kunkli [44]. Their approach estimates the physical properties of the simulated eye by fitting the parameters of a customized eye model to the input monochromatic aberration coefficients.

### Off-axis aberrations

A common limitation of the image-space convolution methods described above is that they ignore the angle of incidence during the PSF calculation and consider the aberrations (and the corresponding PSFs) to be uniform across the entire visual region. This approach is often referred to as the isoplanatic assumption [52, 53]. While such a simplification has several advantages (e.g., input data reduction, faster PSF computation, faster convolution), it fails to provide a complete view of the eye’s actual visual performance.

**Fig. 12:**
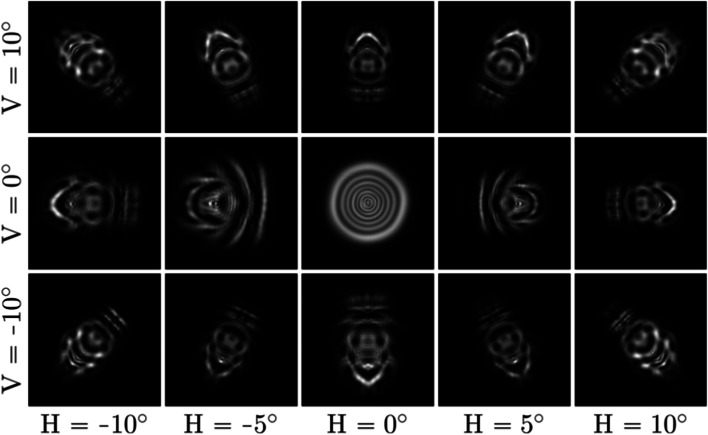
Off-axis PSFs computed for the same object distance

To overcome this issue, Rodríguez Celaya et al. [34] used the ray-tracing approach of Barsky to compute a 3D grid of PSFs, with different axes corresponding to the horizontal angle, vertical angle, and object distance. An example of an off-axis PSF grid for a single object distance is shown in Fig. 12. During the simulation, the per-pixel PSF from the grid was approximated via trilinear interpolation, using the neighboring eight PSFs.

The algorithm of Rodríguez Celaya et al. was later extended by Gonzalez Utrera [42] to support chromatic aberration. In addition, a more sophisticated interpolation algorithm was proposed in this study to split the visual field into several 2 × 2 interpolation regions. The corresponding four PSFs of each region were then used to construct a set of basis functions for a more efficient computation of the PSF at an arbitrary point inside the region.

### Simple kernel approximations

Similar to ray tracing, the computation time for convolution-based approaches can be reduced by confining the simulation to certain types of aberrations. Such approaches typically employ a simplified kernel that only approximates the true PSF and displays some favorable properties that enable a more efficient convolution.

#### Repeated filtering

Rokita [32] proposed the use of a simple 3 × 3 kernel that focuses most of the energy on the central pixel. This kernel is then used to repeatedly filter the input image with the number of iterations determined using the per-pixel radius of the blur circle, which is typically referred to as the circle of confusion (CoC).

#### Uniform elliptical disks

One of the main issues with the repeated-filtering approach is the poor mapping to modern GPUs. This is because current GPUs are severely affected by the speed of memory transactions, and repeated filtering requires a significant number of passes to produce a result for highly defocused images. Consequently, most modern algorithms employ larger kernels that involve only a small number of render passes.

Barbero and Portilla [40] approximated the PSF using a uniform elliptical disk. For each output pixel, the result is produced by taking a fixed set of samples from the neighborhood, based on the corresponding blur ellipse. The parameters of the ellipse (major and minor radii and angle between the major axis and abscissa) are calculated on a per-pixel basis. This process is also performed for each channel of the RGB input, allowing the simulation of chromatic effects.

#### Gaussian kernels

Although a single-pass filtering approach has significant benefits over several considerably small passes, the requirement for a large number of samples still poses some limitations on the extent of possible blurring at a reasonable speed. In computer graphics, this problem is typically solved by using separable kernels [54–56], which are functions that can be computed as the product of lower-rank kernels, to reduce the complexity of the convolution from quadratic to linear.

**Fig. 13:**
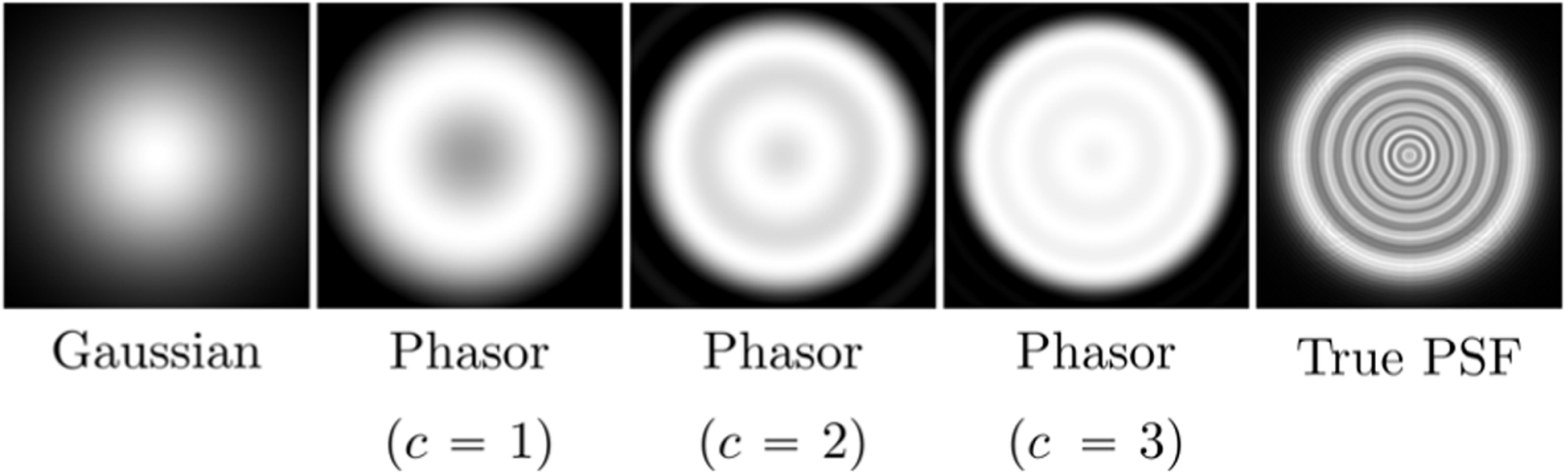
Separable PSF approximation strategies. $c$ denotes the number of complex phasors used to construct the kernels

One of the most well-known separable kernels is the Gaussian kernel, which was used by Tang and Xiao [39] as a substitute for the true PSF of the eye. A comparison of an example PSF and its Gaussian approximation is shown in Fig. 13.

The approach of Tang and Xiao utilizes a small neural network to calculate the radii of the blur ellipse for each input pixel, which they refer to as the blur distribution function (BDF). They employ a schematic eye model to build a custom training dataset via ray tracing. The neural network uses the focal length, pupil size, object distance, and angles of incidence as input, and thus, their algorithm naturally supports off-axis aberrations.

#### Complex phasors

One of the biggest drawbacks of Gaussian kernels is their significant deviation from the true PSF. To overcome this issue, the use of complex phasors has been proposed as a separable filtering approach [57] for DOF simulations. The PSF is approximated as a linear combination of a set of complex-valued basis functions with the number of terms included being application-dependent; 1–3 components are fairly typical, as shown in Fig. 13 for a representative PSF. Although the computational cost of complex phasors is higher than that of the simple Gaussian-filtering approach, complex phasors can produce outputs that much better approximate convolution with the true PSF.

Complex phasors can also be used for vision simulation, as demonstrated by Csoba and Kunkli [43]. Their approach computes the PSF of the simulated eye using the Fraunhofer diffraction formula and fits an ellipse to the resulting PSF. Vision is then simulated using a stretched and rotated version of the original complex kernel, which enables the simulation of low-order visual aberrations and achieves a higher accuracy than Gaussian kernels.

### Partial occlusion

**Fig. 14:**
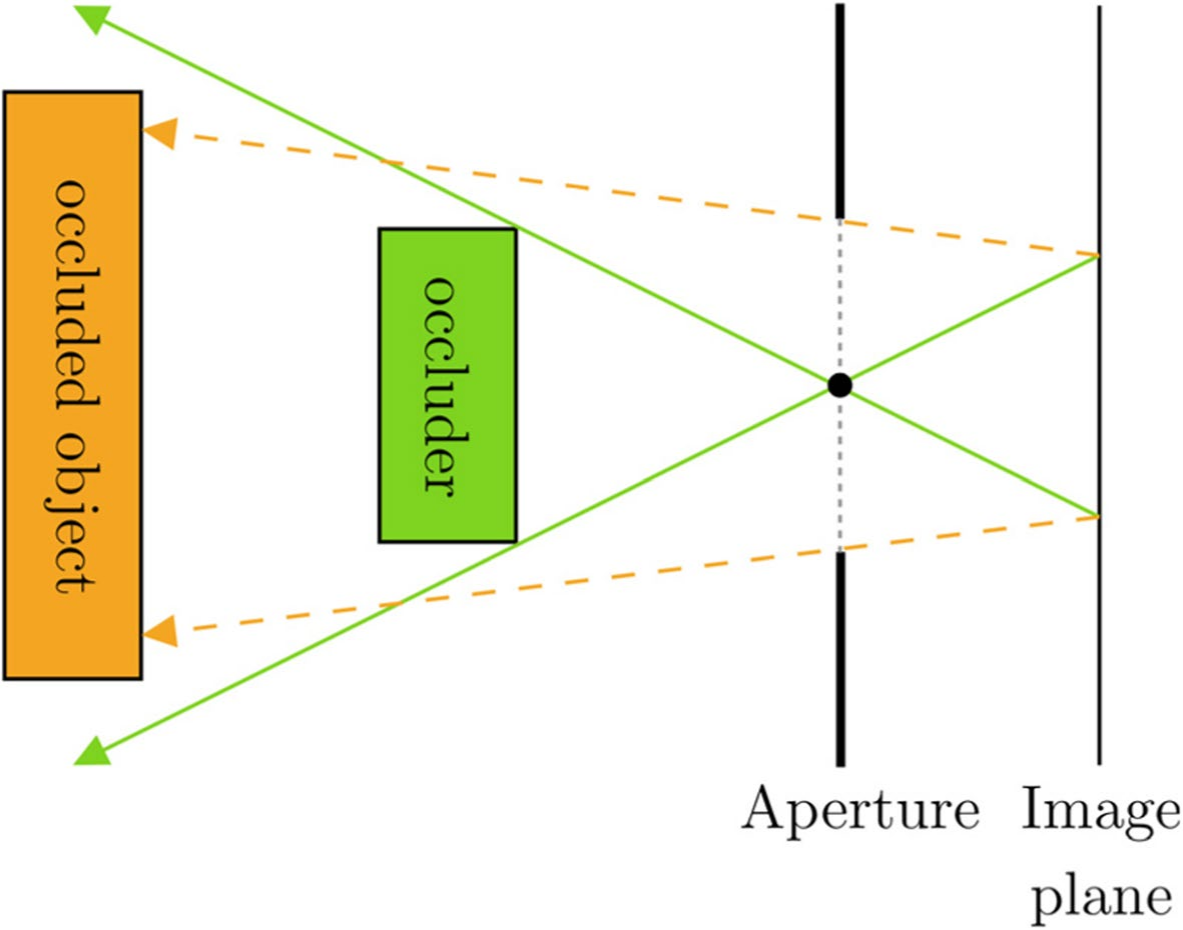
Overview of partial occlusion. Rays are unable to reach the image plane when a pinhole camera model is used (green). Parts of the occluded object are reachable by considering the physical extent of the lens (orange)

Image-based vision simulation algorithms are typically implemented as a postprocessing filter for input images that were originally rendered using pinhole camera models. This approach results in a one-to-one mapping between object space and image space, which fails to properly account for the partially covered object points that are invisible on the pinhole image. The problem is visualized in Fig. 14.

This issue is typically solved by using layered inputs [51, 58] to provide the missing background information. Lima et al. [45] used a similar layered method to simulate low-order aberrations of the eye. This approach places a regularly spaced grid of samples on the pupil for each output pixel. They then utilize a tree data structure to precompute the list of relative pixel locations across the layers that contribute to each unique grid location on the pupil. The final result is produced by traversing the tree for each pupil sample, accumulating individual samples.

### Multiview synthesis

**Fig. 15:**
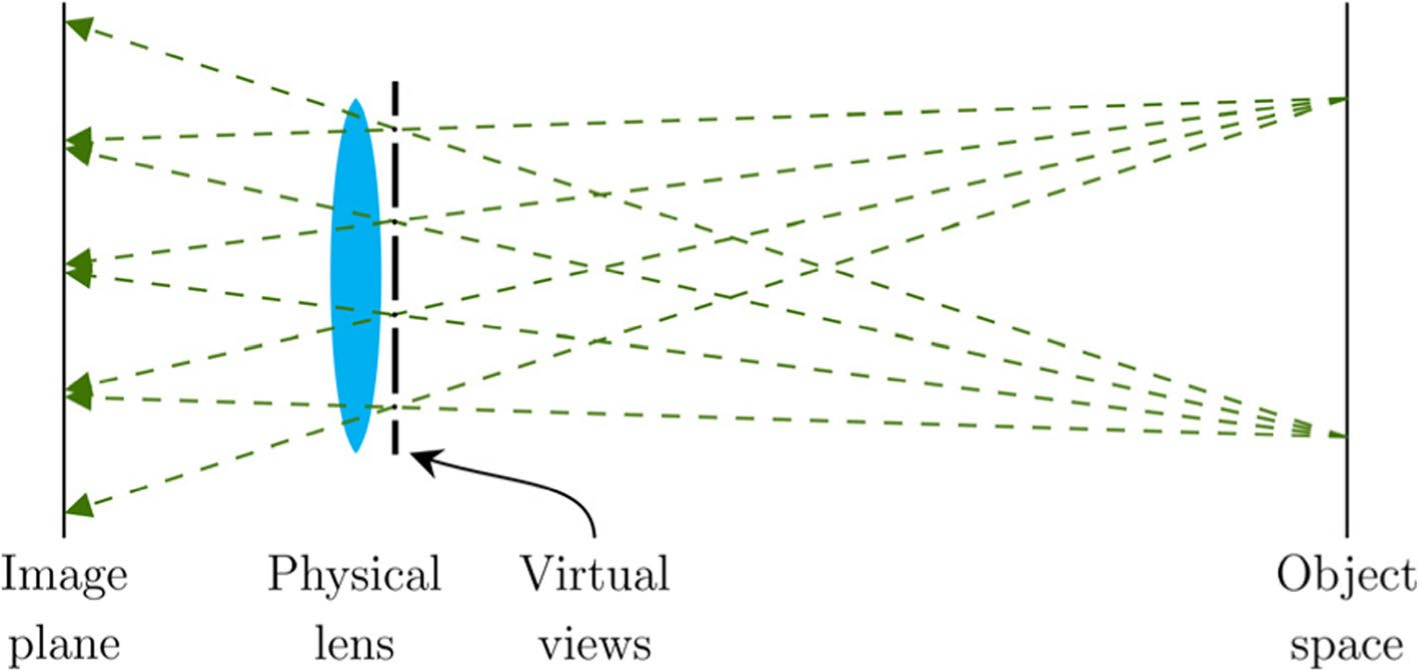
Multiview image synthesis. The physical lens is sampled to construct several different viewpoints. The scene is then rendered from each view and accumulated into a single image

A different approach for simulating distributed effects is to place virtual viewpoints on the pupil and render the scene from each viewpoint using rasterization. This technique, often referred to as the accumulation-buffering method, was introduced by Haeberli and Akeley [59]. A schematic of this technique is shown in Fig. 15. A common limitation of this approach is that smooth high-resolution outputs require a prohibitively large number of views to be rendered.

To simulate vision using accumulation buffering, Kakimoto et al. [35] proposed the use of a 3D blur field that describes the amount of blurring produced by the eye at a specific location in the scene for a given sample on the pupil. They demonstrated the applicability of wavefront tracing [35] and conoid tracing [36] for precomputing the blur field. Both approaches attempt to improve the computation speed of traditional ray tracing by considering only the higher-level properties of the induced wavefronts. During the simulations, a unique view is constructed for each pupil sample. These views are rendered by applying an offset to each vertex of the triangular scene, which was determined by sampling the blur field at the world-space location of the vertex. The final vision simulation is then produced by accumulating the different views into a single output image.

## Discussion

In previous sections, the working principles of the most common approaches for simulating aberrant human vision were described. In this section, several of the most important properties, which can guide the selection of the correct approach for a given problem, are discussed and compared. An overview of these properties is provided in Table 3. In the remainder of this section, a detailed discussion on the properties of the algorithms described in this survey is presented.

**Table 3:** Comparison of several important properties of the selected works discussed in this study

**Method**	**Required eye data**	**Pupil size variability**	**Focus distance variability**	**Input scene data**	**Peripheral vision**	**Partial occlusion**	**Real-time performance**	**Inherent eye aberrations**	**High-order aberrations**	**Chromatic aberration**	
Mostafawy et al. [9]	ES + CRZ	ES	ES	3D	✔	✔	✘	✔	✔	✘	Ray tracing
Fink and Micol [10]	ES	ES	ES	I	✔	✘	✘	✔	✔	✘	
Wu et al. [11]	ES	ES	ES	3D	✔	✔	✘	✔	✔	✘	
Wang and Xiao [12]	SL	FA	FA	3D	✘	✔	✘	✘	✘	✘	
Wei et al. [13]	ES	ES	ES	3D	✔	✔	✘	✔	✔	✔	
Dias et al. [14]	ES	ES	ES	3D	✔	✔	✘	✔	✔	✘	
Cholewiak et al. [15]	FL	FA	FA	3D	✔	✔	✘	✘	✘	✔	
Lian et al. [16]	ES	ES	ES	3D	✔	✔	✘	✔	✔	✔	
Vu et al. [17]	ES	ES	ES	I	✔	✘	✘	✔	✔	✘	
Camp et al. [30]	CT	AM	X	I	✘	✘	✘	✘	✔	✘	Image space
Greivenkamp et al. [31]	ES	ES	ES	I	✘	✘	✘	✔	✔	✔	
Rokita [32]	FL	FA	FA	I + D	✘	✘	✔	✘	✘	✘	
Barsky [33]	WA	AM/AC	AM	I + D	✘	✘	✘	✔	✔	✘	
Rodríguez Celaya et al. [34]	PL	X	X	I + D	✔	✘	✔	✘	✔	✘	
Kakimoto et al. [35]	PL	FA	FA	3D	✔	✔	✔	✔	✘	✔	
Kakimoto et al. [36]	PL	FA	FA	3D	✔	✔	✔	✔	✘	✔	
Barsky [37]	WA	AM/AC	AM	I + D	✘	✘	✘	✔	✔	✘	
Watson [38]	WA	AC	AM	I	✘	✘	✘	✔	✔	✔	
Tang and Xiao [39]	ES	ES	ES	I + D	✔	✘	✔	✔	✘	✘	
Barbero and Portilla [40]	PL	FA	FA	I + D	✔	✘	✘	✘	✘	✔	
Cholewiak et al. [41]	WA	FA + AM/AC	FA + AM	I	✘	✘	✘	✔	✔	✔	
Gonzalez Utrera [42]	PL	ES	ES	I + D	✔	✘	✔	✘	✔	✔	
Csoba and Kunkli [43]	SL	FA	FA	I + D	✘	✘	✔	✔	✘	✘	
Csoba and Kunkli [44]	WA	ES	ES	I + D	✘	✘	✔	✔	✔	✔	
Lima et al. [45]	SL	FA	FA	I + D, L	✘	✔	✔	✔	✘	✔	
	Control			Rendering				Aberrations			

Possible values for required eye data: *FL* Focal length, *ES* Eye structure parameters, *CRZ* Corneal refractive zones, *SL* Spectacle lens, *PL* Progressive lens, *CT* Corneal topography measurements, *WA* Wavefront aberration information

Possible values for pupil size variability and focus distance variability: *ES* Structural changes to the eye model utilized, *FA* Adjustments to mathematical formulations such as the recomputation of the focal length, *AM* Additional clinical measurements, *AC* Aberration conversion formulas

Possible values for input scene data: *I* A single 2D image or rendering, *I* + *D* A 2D image with additional depth information, *I* + *D, L* Several layers of 2D color and depth images, *3D* 3D scene representation such as 3D triangles

### Input data and personalization

One of the key differentiators between ray-tracing and image-space approaches is the required input data. Most image-space approaches rely on PSFs, which are comparatively simple to acquire using a wavefront aberrometer, as demonstrated in several recent studies. Furthermore, wavefront aberrations are typically described using the well-understood and standardized approach of Zernike coefficients [60], which can be obtained from aberration databases or even computed from spectacle lens prescriptions using conversion formulas [61].

Ray-traced vision simulation algorithms, however, require a complete eye model to support arbitrary visual aberrations. Such a description can be obtained by utilizing an existing schematic eye model, but the uses of such models are limited because they are not representative of the individual’s eye structure. It is possible to extend the schematic eye model using additional optical elements to simulate glasses; however, this approach is still not ideal if the true physical characteristics of the simulated eye are important. Taking measurements for all required eye parameters is a viable alternative and is often performed in population studies (such as in ref. [62]). However, this process requires several devices and measurements, reducing the reliability of the approach.

Recently, the use of an optimization-based eye-structure estimation process was proposed by Csoba and Kunkli [44] as an effective means of overcoming these limitations. A custom parametric eye model was used with an optimization procedure to find a set of eye parameters that would approximate the input aberration coefficients suitably. Such an approach facilitates the use of wavefront aberrations as input to the simulation but also provides a usable description of the entire eye structure as the output of the process. Consequently, eye structure estimation preserves the best characteristics of the two approaches, with its main disadvantage being the computation time.

### Dynamic eye states

**Fig. 16:**
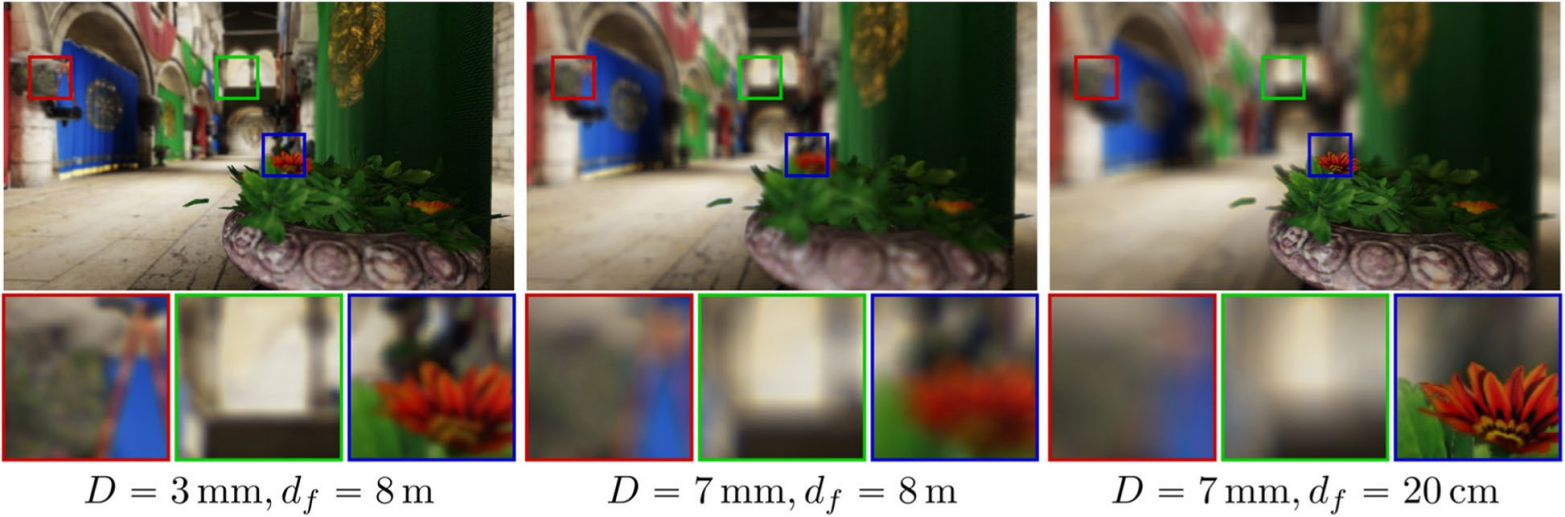
Showcasing the effects of varying pupil sizes ($D$) and focus distances (${d}_{f}$) on vision

The behavior of the eye with varying pupil sizes and focus distances is also of interest. While exploring the performance of the eye in one state is tremendously useful in itself, aberrations vary greatly as the pupil diameter or state of accommodation changes. We also demonstrate the effects of changing the pupil diameter and focus distance in Fig. 16 on an example scene. Accounting for these dynamic parameters can provide a more holistic view of the overall performance of the eye and is crucial for studying the different scenarios that occur in the everyday life of a patient.

Handling a variable pupil size is trivial for all the approaches presented in this study. First, the pupil diameter is already a part of the model when the physical eye structure is used. Second, methods that rely on the CoC include the diameter in the definition of the CoC. Finally, image-space approaches that use Zernike coefficients to calculate the PSF can use scaling formulas to obtain the corresponding Zernike coefficients [38].

Refocusing, however, is a significantly more difficult problem. During accommodation, the shape of the crystalline lens changes drastically. As a result, even if the full physical structure of the relaxed eye is available, only the exact physical properties of the focused lens can be estimated. This issue is even more severe for algorithms that rely on wavefront aberrations because these algorithms do not possess any information about the shape of the eye’s optical elements. Consequently, the only viable approaches that we are aware of are extra aberration measurements and eye structure estimation (as described in input data and personalization subsection).

### HOA

**Fig. 17:**
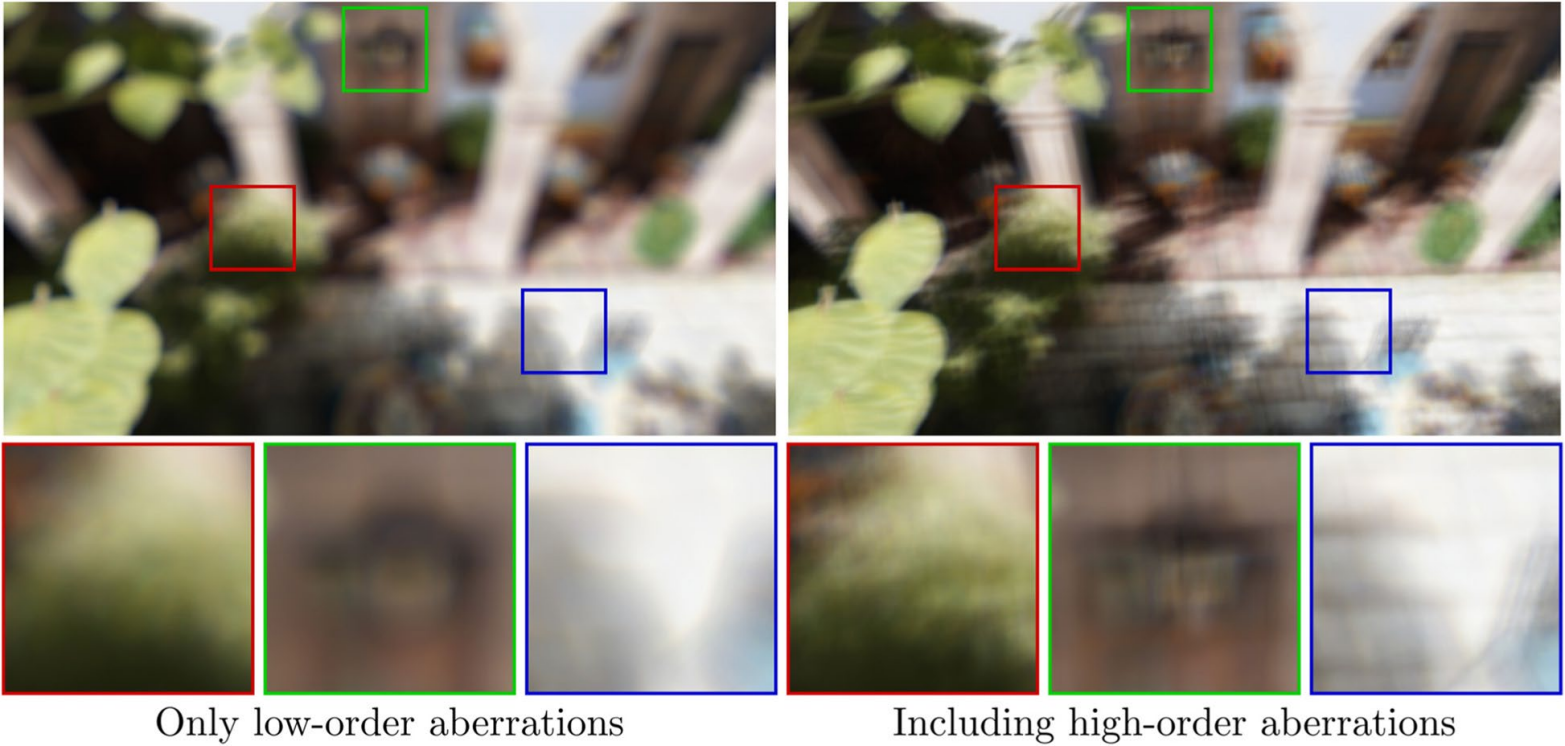
Comparing the impact of simulating HOA. Low-order aberrations are the same in both simulations. Omitting HOA produces a substantially different output and thus gives an incorrect representation of the individual’s vision

HOA have a significant impact on the overall quality of vision [63], as shown in Fig. 17. Several factors affect the HOA of the eye, with corneal deformation and internal optical elements being common causes [64].

The complex origin of HOA hinders ray-tracing methods from being able to capture HOA when the full eye structure is not used. Furthermore, the deformation of the cornea requires special treatment even if a complete eye model is used, as a single aspherical element lacks the flexibility to represent the arbitrary deformations of a real cornea.

The issue is less severe for image-space approaches because including HOA in the PSF is easily accomplished using Zernike coefficients. However, the same cannot be said about the various approximation approaches (the Gaussian kernel and complex phasor algorithms), as the kernels that they employ are specifically chosen to approximate low-order aberrations (mostly, defocus and astigmatism).

### Chromatic aberration

**Fig. 18:**
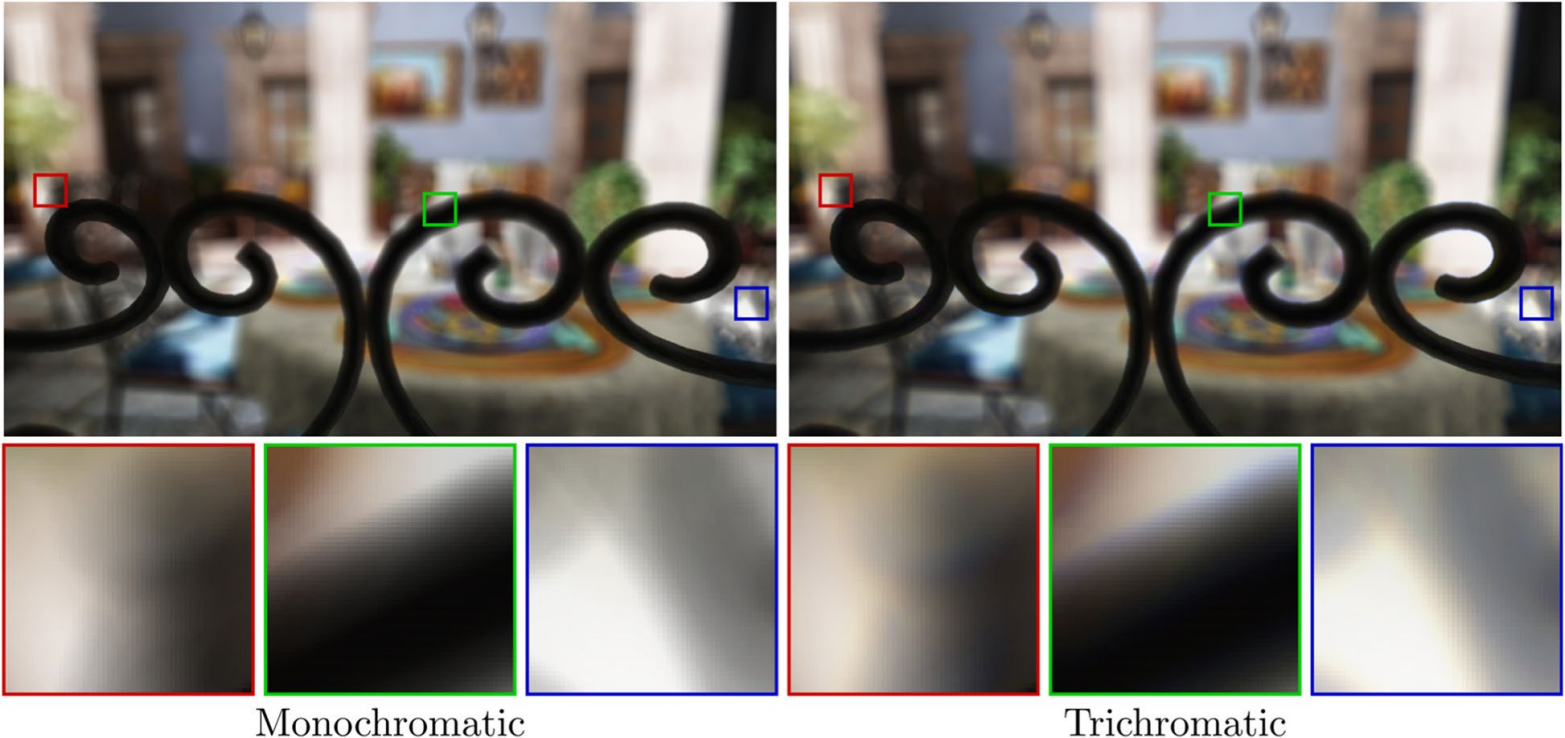
Demonstrating the impact of chromatic aberration. Although a monochromatic simulation is a good representation of the overall visual acuity, convolution with the wavelength-dependent PSF more accurately simulates the true eye performance

Another crucial aspect of aberrations is their wavelength-dependent nature. As demonstrated by Cholewiak et al. [15], chromatic aberration is an important factor in the accommodation process. Therefore, simulating chromatic aberration is essential in reducing the vergence-accommodation conflict that typically occurs in virtual reality (VR) approaches. The effect of chromatic aberration is demonstrated in Fig. 18, where vision simulations are compared using monochromatic and trichromatic PSF models.

As described in chromatic aberration subsection (in vision simulation section using ray tracing), simulating chromatic aberration is trivial with ray tracing if a full eye model is employed. The ray-tracing process can be extended by introducing a wavelength dimension and sampling, to correctly model the full spectrum. However, even without the full-eye model, it is possible to acquire correct simulations using the simplified ray-tracing approach of Wang and Xiao [12], which accounts for the wavelength-dependent focal length of the eye—as demonstrated by Cholewiak et al. [15].

Regarding image-space approaches, simulating chromatic aberration is equivalent to using a wavelength-dependent PSF kernel. The main problem is the calculation of the necessary Zernike coefficients, which can be solved by using a conversion formula—as demonstrated by Watson [38]. However, kernel-approximation approaches typically ignore chromatic aberration. The understanding of the authors is that this simplification is a result of their performance-focused nature and not an inherent limitation of the approaches because when the different channels of the input image are treated separately it is trivial for these algorithms to handle chromatic aberration.

### Peripheral vision

**Fig. 19:**
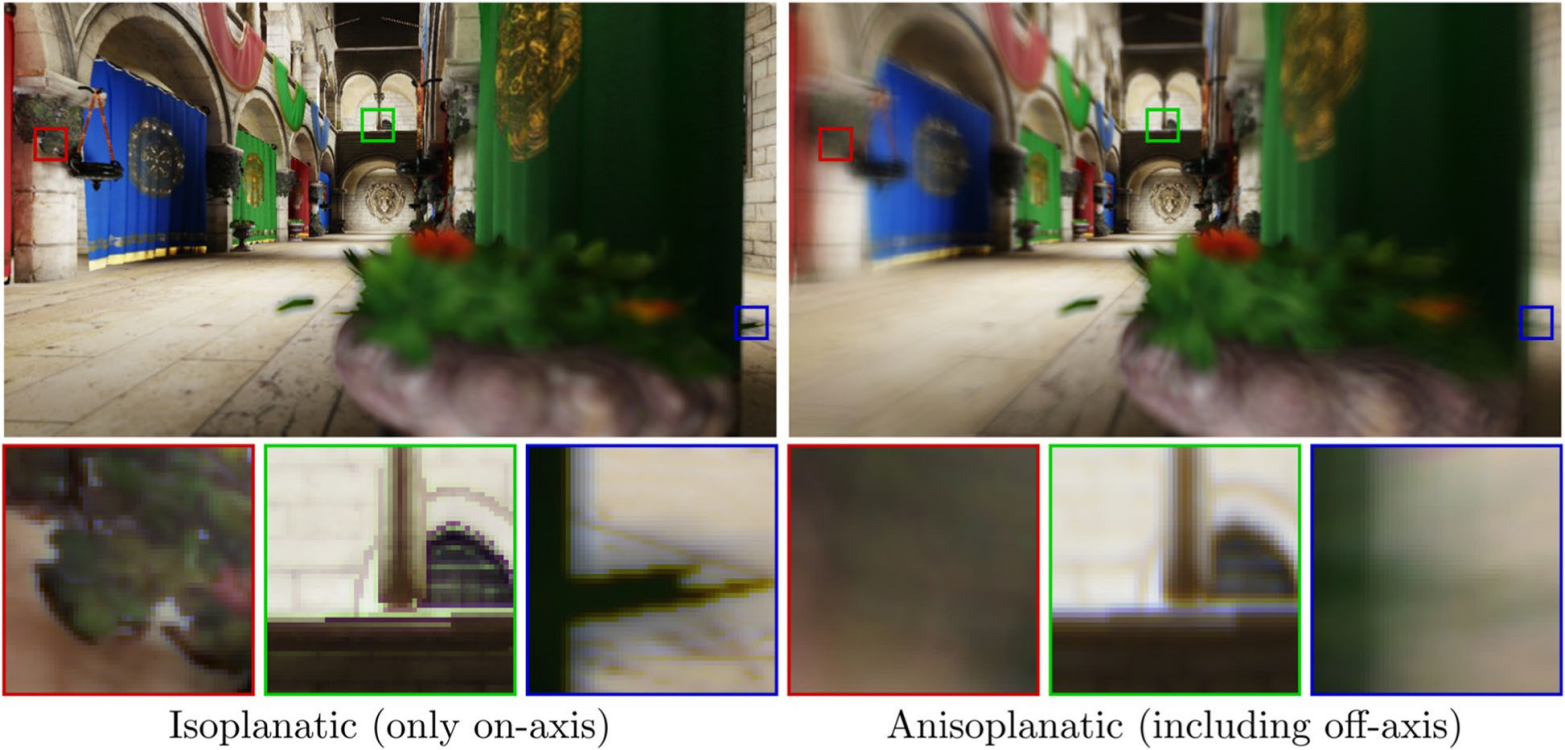
Demonstrating the impact of peripheral vision. The blurriness is the same for the entire visual field when only the on-axis PSF is considered. Correctly modeling the off-axis PSFs provides a more holistic view of the simulated vision

Many existing aberration simulation techniques focus on the central area of the visual field and only attempt to simulate on-axis aberrations of the eye. Such an approach is often referred to as isoplanatic approximation, and the way it works is described by Barsky [33]—when the eye moves toward the object to be focused, it automatically aligns the focused object with the visual axis. However, the information acquired through the off-axis area (which can be simulated using anisoplanatic techniques) still possesses important cues for the human brain, as demonstrated by several studies that focus on the loss of peripheral vision [65, 66]. We also demonstrate the importance of peripheral vision by comparing examples of isoplanatic and anisoplanatic rendering in Fig. 19.

When using physically accurate ray tracing and a complete eye model, the simulation of off-axis vision is a natural result of the rendering approach. Furthermore, the outputs are accurate as well, provided the correct retina shape is used. However, it is impossible to correctly simulate peripheral vision using a simplified ray-tracing model.

To simulate off-axis aberrations using PSF-based convolution, first the off-axis PSFs must be obtained and subsequently, the corresponding aberration descriptions. Similar to refocusing, these aberrations vary heavily from person to person and depend greatly on the physical properties of the eye. Thus, the only way of obtaining these coefficients is through measurements and eye structure estimation. However, even if the off-axis aberrations are known, very few PSF-based algorithms are constructed with these considerations. Among the algorithms described in this paper, per-pixel interpolation of the PSF grid is the only approach that is capable of handling the spatially varying off-axis PSF.

Finally, vision simulation algorithms that are based on PSF approximation can more easily support off-axis vision because such approaches only need to obtain the per-pixel CoC. To this end, the BDF-based approach of Tang and Xiao [39] can be used as an efficient means of computing the CoC values during rendering. However, the BDF is built using ray-traced data, and the physical structure of the eye is required for actual patient data, the acquisition of which can be performed using parameter measurements of the eye structure that are estimated from wavefront aberrations of the eye. Furthermore, as described earlier, these methods are unable to properly capture the exact shape of the PSF, and thus, they are mostly useful only for simulating the degree of peripheral blurring.

### Performance

Another critical aspect of these algorithms is their overall performance. Vision simulation is typically employed as part of a larger system with a specific end goal, and throughput is often a key aspect of these applications. In particular, interactive eye disease simulation, vergence-accommodation conflict reduction, vision testing, aberration-correcting displays, and machine-learning data generators are all applications in which the total computation time of the vision simulation step is a serious limiting factor.

The cost of producing a single image depends significantly on the complexity of the scene owing to the nature of ray tracing. Although more recent algorithms are empowered with massive computational capabilities of the GPUs [13, 17], their authors still report computation times that are inappropriate for interactive applications, even for simple input scenes and comparatively small output resolutions. Currently, consumer-grade hardware is not sufficiently powerful to offset the inherent cost of ray tracing and the large number of rays necessary for a single output pixel. Nonetheless, ray tracing is typically used only for vision simulation when the importance of output quality outweighs the cost of producing a single image.

Image-space algorithms, however, typically display much better performance characteristics. The main factors that contribute to the ray-tracing cost are the large number of per-pixel rays and the complexity of tracing a single ray through the optical system, which are hidden costs in the pre-computation part of convolution. Therefore, convolution-based approaches tend to be substantially faster than ray tracing. Furthermore, they also have the potential to achieve interactive, or even real-time performance provided GPU-based acceleration is available, as demonstrated by multiple recent approaches [39, 44, 45], making these approaches an ideal choice for low-latency, high-throughput applications.

### Scene configurations

Finally, the possible limitations of vision simulation algorithms are discussed with regard to the types of supported input scenes. Owing to the continued efforts of computer graphics and ophthalmology researchers, ray-tracing and convolution-based vision simulation algorithms are now readily available for both 2D and 3D input scenes. The main factor to consider is the performance impact of the input type, as described in the previous section.

The handling of partial occlusions, however, is of particular interest. As the name of the phenomenon suggests, partial occlusion is only relevant to 3D scenes and materializes when a blurry foreground object partially covers the background, as described in partial occlusion subsection. Handling partial occlusion is trivial in using ray tracing because the resulting image is constructed by sampling the full pupil; therefore, with a sufficiently large number of samples, all the relevant parts of the scene contribute to the final value of each pixel.

However, image-space approaches typically struggle with partial occlusion because the pinhole camera model that is traditionally used to render the input only considers rays that pass through the center of the pupil. As described in the partial occlusion subsection, a commonly employed solution to this issue is the use of layered input images to supply the necessary information about the missing background pixels. However, the disadvantage of this approach is the increased cost of rendering and processing extra layers of the input.

## Applications

As mentioned in the introduction, vision simulation algorithms have numerous potential applications [3–6]. In this section, a short introduction on the areas that would benefit from the proper simulation of human visual aberrations is provided.

### Visual acuity metrics and vision testing

One of the earliest uses of visual aberration simulations was the calculation of visual acuity metrics. Such methods utilize computer simulations to generate images corresponding to the vision of an individual. The simulation of visual aberrations is key in these works [31], which is often extended by various neural-processing functions to obtain a more holistic view of the overall acuity of the observer [67, 68]. The resulting simulations are then analyzed using image metrics, such as modulation and Strehl ratio, to evaluate acuity.

Despite the existence of physical devices that carry out such tasks, as noted by Kordek et al. [69], computer-based acuity simulations provide some important advantages over clinical approaches. Simulations are not hampered by optical setup limitations, requiring only a comparatively inexpensive desktop computer, reducing the impact of eyelid squinting.

### Study of the human eye

Computer simulations of human visual aberrations can also be beneficial for studying the mechanisms of the human eye. A typical approach in these types of experiments is to construct computer models and derive metrics based on specific image characteristics, visual acuity, or aberrations, using a digital model. In addition, the results can be compared to data obtained from real-world measurements performed on the human eye, which are tremendously useful for testing the validity of hypotheses about the human eye. For example, Tabernero et al. [70, 71] used a similar approach to study the mechanisms of aberration compensation in the eye. The aberrations of digital eye models were computed using data acquired through aberrometer measurements. The use of computer-based ray-tracing tools is essential for obtaining these results.

Most of the existing studies are limited to analyzing only the aberration structure of the eye, whereas utilizing vision-simulated imagery can lead to important new theoretical results on the mechanisms of human vision, providing a deeper understanding of the human visual system and its deficiencies. For example, Cheng et al. [72] used a similar approach to study the impact of HOA on low-order refractive errors. Through-focus vision simulations were used in the presence of visual aberrations to calculate several acuity metrics. The computation of vision-simulated imagery was essential in facilitating this study.

Another area where vision-simulated imagery can be highly useful is the visualization of eye diseases in virtual environments. These experiments typically utilize vision simulations to determine an individual’s ability to perform certain tasks in the presence of reduced vision. Using this approach, experts can gain a better understanding of the impact of such conditions on the everyday lives of affected individuals, and thereby facilitate the development of proper tools and devices that better support the lives of these people. To this end, synthetic [61, 73–77], VR [78–80], and augmented reality (AR) [80–82] environments have already been successfully used.

### Surgery and lens planning

Simulating visual aberrations of the eye can be tremendously important for invasive processes, such as intraocular lens implants [83] and laser surgery [84]. These procedures have a substantial impact on eye performance and often introduce undesired visual aberrations. Vision simulations can reduce the likelihood of post-surgery patient dissatisfaction by facilitating the inclusion of patient-specific information during the planning process. Instead of relying only on numerical metrics, the use of vision-simulated imagery of an individual’s pre- and post-surgical vision can be greatly beneficial.

Another common form of vision correction is the use of progressive addition lenses (PALs). Corrective spectacle lenses are widely used to treat presbyopia (the gradual loss of focusing ability on near objects), which is a common age-related condition. The main differentiating property of these lenses from traditional spectacle lenses is the continuous change in optical power over their surfaces, which significantly increases the complexity of designing a lens that properly treats the vision of the individual. Vision simulation methods are a quick and inexpensive way to evaluate the performance of various PAL designs in virtual environments [34, 42, 85–87]. The success of the procedure can be further improved if the knowledge of the user’s visual aberrations can be included in the simulation. Utilizing vision-rendering techniques can significantly improve the fit of the resulting progressive lenses and provide better user satisfaction.

### Vision-correcting devices

Non-invasive vision-correcting devices have recently made significant progress, and vision simulation is also a crucial element for their development. Among the earliest devices to be developed, vision-correcting displays typically apply inverse blurring to the displayed imagery to compensate for the aberrations of the observer of the display [88–90].

Image-prefiltering approaches often suffer from contrast issues and ringing artifacts. One way of addressing these problems is by utilizing multilayer displays [91, 92] such that multiple semi-transparent layers are stacked on top of one another, with each displaying a different prefiltered image. The key aspect of this approach is selecting the filtering to maximize the contrast of the combined resulting image.

Light-field displays have also been proposed as an alternative to overcome the limitations of single-image solutions, whereby a microlens array is placed on top of the display to provide a higher degree of control over the directionality of the emitted light. Vision correction can be achieved using such displays [93–95] by utilizing vision simulations to prefilter the displayed images.

With recent advancements and increased accessibility of optical see-through head-mounted displays (OST-HMDs), vision correction using these devices has become a potential application area [96]. A camera attached to the OST-HMD is used to capture scene information. This image is then filtered using vision simulation methods to obtain an ideal image that an emmetropic eye would see. In the last step, the resulting image is further processed using aberrations of the wearer’s eye to display an image on the virtual screen that is aberration-free.

Recently, holographic near-eye display systems [97] have been used for vision correction. These devices operate by placing in front of the viewer a holographic display that modulates the amplitude and phase of the incoming light. Vision simulation methods are crucial for computing the modulations necessary to correct an individual’s aberrations. However, they can also be used for device calibration [98].

### Reducing discomfort from wearing extended reality headsets

XR) displays are also a topic of active research and are heavily influenced by the human visual system. A typical problem with such systems is the inconvenience and fatigue experienced by the user, often because of the disparity between the displayed synthetic imagery and the eye’s natural vision. Enhancing simulated renderings with cues, such as chromatic aberration [15, 41] and depth-dependent blur [32, 99–101], can help alleviate this disparity. These techniques attempt to enhance the presented imagery with aberrations that are natural to the wearer’s vision, using vision simulation and considering the inherent visual aberrations of the user to achieve the best possible results.

Fatigue caused by visual disparity is not the only source of discomfort in these devices. Individuals with aberrant vision are often required to wear corrective glasses while using XR headsets, which severely degrades the overall experience of the user. Through the application of vision simulation, the presented imagery can be preprocessed with the inverse aberrations of the wearer, similar to certain vision-correcting displays. Such an enhancement of head-mounted devices eliminates the need to wear glasses, facilitating their use and further improving the convenience of users [102].

Another common form of inconvenience associated with AR displays is user fatigue. Users are often required to rapidly switch their cognitive attention and eye accommodation between virtual and real information, which can lead to significant fatigue. Recently, Arefin [103] used vision simulation to design a special-purpose font to reduce the fatigue originating from these sources when using AR displays.

### Training virtual humans

Another area where vision simulation is highly useful is the training of autonomous virtual humans. To ensure that the behavior of a virtual human closely matches that of a real person, it is essential to accurately mimic all aspects of the human visual system. Because visual aberrations play significant role in vision and affect each individual, incorporating them into the simulation pipeline is necessary to achieve the desired results. Therefore, Nakada et al. [104, 105] utilized a ray-tracing approach for the optical simulation of vision. With the advancements and availability of a wide range of vision simulation approaches, we believe that this area could greatly benefit from future experiments with other types of algorithms.

## Conclusions

In this study, several approaches for simulating human vision that are affected by wavefront aberrations have been described. These methods were categorized as either based on ray tracing or image-space convolution, depending on the principal tools employed. Ray-tracing methods utilize a wide range of different physical eye models and distributed ray tracing to produce the final results; whereas, convolution-based techniques employ ray tracing or wavefront aberrations to compute the PSF of the eye, which is then used as the kernel for convolution. Based on a description of the various approaches, we also compared several important characteristics, which can serve as guidelines in selecting the correct algorithm when solving a problem that involves the simulation of human vision aberrations. Finally, we outlined several important application areas where simulation of human vision and visual aberrations play an essential role.

Despite the large number of available techniques, there is still room for further research in this field. In particular, performance is a critical area where improvements are always welcome. With increased throughput, ray-tracing algorithms can become a viable alternative to convolutional approaches in real-time environments, particularly in case of more complex eye models. The significant recent advancements made in the field of dedicated ray-tracing hardware accelerators and neural network-based approaches, such as reconstruction from low sample-count renderings [106], could be useful in achieving these goals. However, the length of precomputation and the cost of including peripheral vision and partial occlusion are areas where further research would be beneficial for image-space methods. Efficient means of computing human PSFs and carrying out convolution, such as in the Laplacian domain [107], are essential in alleviating these issues. Finally, patient-specific information is seldom utilized in existing studies because data acquisition is a severely limiting factor. Additional methods that specifically tailor the simulation to the individual (such as the eye structure estimation approach proposed by Csoba and Kunkli [44]) could considerably improve the personalized usability of vision simulation algorithms.

Visual aberrations play an important role in our everyday lives, and with recent advancements in fields such as aberration-correcting devices, head-mounted displays, and XR devices, perfected vision simulation tools are essential to pave the way for future progress in these areas.

## Figures and Tables

**Fig. 20:**
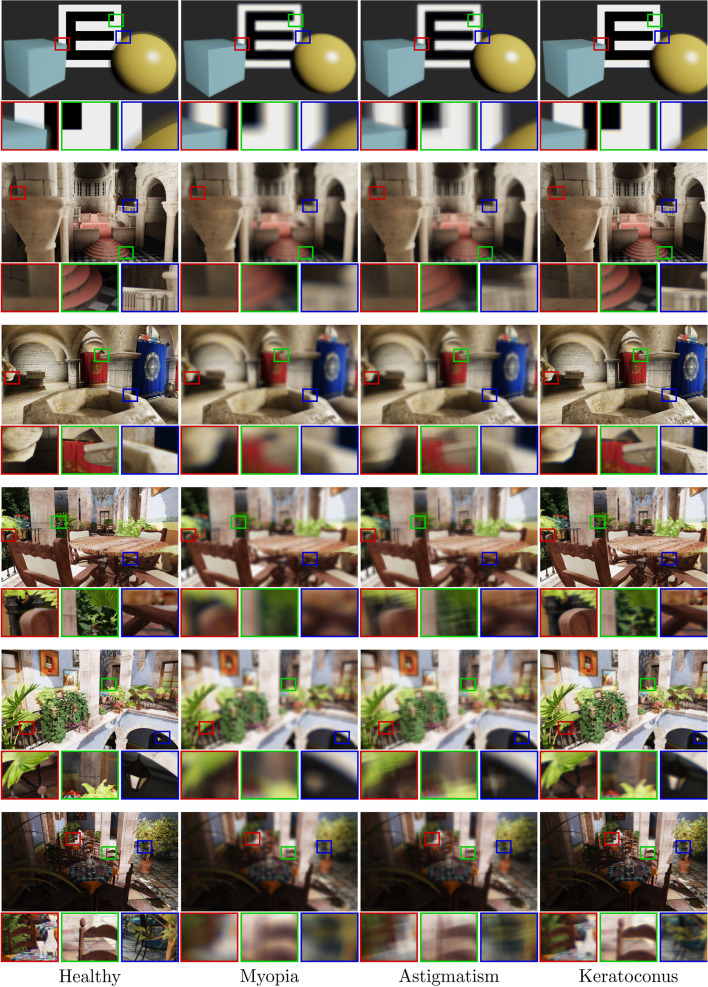
Vision-simulated renderings for four different eye conditions and three different test scene configurations

## Data Availability

Not applicable.

## Appendix

### Additional vision-simulated renderings

In this study, additional simulations were performed for several different test scenes and eye conditions. The resulting renderings, displayed in Fig. 20, compare the vision of a healthy eye with those suffering from myopia, astigmatism, and keratoconus, in a variety of scenes. All images were rendered by focusing on the far plane with a 7 mm pupil size. Similar to all vision-simulated renderings presented in this study, these images were generated using PSF-based convolution, utilizing the ENZ diffraction theory to generate the necessary PSFs.

## Abbreviations

DOF: Depth-of-field
HURB: Heisenberg uncertainty ray bending
PSF: Point-spread function
OSPSF: Object-space point-spread function
ENZ: Extended Nijboer-Zernike
GPU: Graphical processing unit
CoC: Circle of confusion
BDF: Blur distribution function
HOA: Higher-order aberrations
VR: Virtual reality
AR: Augmented reality
PAL: Progressive addition lens
OST-HMD: Optical see-through head-mounted display
XR: Extended reality
